# Exploring RPA1-ETAA1 axis via high-throughput data analysis: implications for PD-L1 nuclear translocation and tumor-immune dynamics in liver cancer

**DOI:** 10.3389/fimmu.2024.1492531

**Published:** 2024-11-26

**Authors:** Gaofeng Qin, Zengkuan Chen, Weihong Tian, Hongbo Chen, Yu Zhang, Wangzhi Wei

**Affiliations:** ^1^ Liaoning Technology and Engineering Center for Tumor Immunology and Molecular Theranotics, Collaborative Innovation Center for Age-related Disease, Life Science Institute, Jinzhou Medical University, Jinzhou, Liaoning, China; ^2^ College of Basic Medical Science, Jinzhou Medical University, Jinzhou, Liaoning, China; ^3^ Department of Intensive Care Medicine, Yingkou Central Hospital, Yingkou, Liaoning, China

**Keywords:** replication protein A (RPA), Ewing tumor-associated antigen 1 (ETAA1), PD-L1, immune infiltration, liver cancer

## Abstract

**Introduction:**

ETAA1 is recruited to DNA damage sites via its RPA -binding and ATR -activating domain (AAD) motifs, where RPA binding is crucial for ETAA1’s regulation of ATR activity.

**Methods & results:**

Our findings associate Programmed Death- Ligand1 (PD-L1) with the RPA1-ETAA1 axis, suggesting that upregulated RPA1 -dependent ETAA1 may facilitate PD-L1 nuclear accumulation. We observed strong correlations between ETAA1 and RPA1 with the components involved in HDAC2-mediated deacetylation, clathrin -dependent endocytosis, and PD-L1 nucleocytoplasmic shuttling, aligning with the established regulatory pathway of PD-L1 nuclear translocation. Moreover, nuclear PD-L1 transactivates a panel of pro-inflammatory and immune response transcription factors, potentially reshaping the tumor immune microenvironment. We identified a landscape of infiltrating lymphocytes influenced by ETAA1, finding that levels of ETAA1 were negatively correlated with CD8^+^ T and Natural Killer T (NKT) cells, but positively correlated with CD4^+^ T helper 2 (Th2) cells, cancer-associated fibroblasts (CAFs), myeloid-derived suppressor cells (MDSCs), neutrophils and regulatory T cells (Tregs), suggesting a potential role in immune evasion. Further analysis shows that the RPA1-ETAA1 axis is significantly associated with multiple metastasis mediators and unfavorable liver cancer progression, with higher expression observed in advanced stages and poorly differentiated subgroups.

**Discussion & conclusion:**

These findings expand the role of the RPA1-ETAA1 axis beyond DNA repair, highlighting its potential as a target for cancer therapy.

## Introduction

Late-stage hepatocellular carcinomas present substantial treatment challenges, leading to increased cancer mortality (1). Surgical intervention is viable for only a small fraction of early-stage patients. In advanced cases, treatment options typically include trans-arterial chemoembolization (TACE) (1), external beam radiation therapy (EBRT) (2) and the combination of sorafenib with radiotherapy (3). However, these treatments often encounter issues such as DNA damage, drug toxicity, and limited effectiveness (1). Immunotherapies, including the Programmed Cell Death 1 (PD-1) inhibitor nivolumab, either alone or in combination with the CTLA-4 inhibitor ipilimumab, have been explored for liver cancer (1, 4). Unfortunately, the emergence of multi-drug resistance frequently leads to metastasis and recurrence, underscoring the necessity for further investigations to develop more effective strategies, particularly those targeting tumor invasion and enhancing immune surveillance.

One such mechanism involves the PD-1/PD-L1 pathway. Membrane-anchored PD-L1 on tumor cells is well recognized for its engagement with PD-1 on T cells, allowing the tumor to evade anti-tumor immunity (5). Upon shedding of the extracellular domain, the active C-terminal PD-L1 fragment translocates into the nucleus (6). The accumulation of nuclear PD-L1 in tumor cells promotes the expression of multiple pro-inflammatory and immune response genes, suggesting a potential link between tumor aggressiveness and PD-L1 translocation (6, 7). Additionally, exogenous cellular stress induces PD-L1 upregulation in cancer (8), while ATR -mediated DNA repair signaling pathways further enhance the PD-1/PD-L1 axis (9). This up-regulation functions as an inhibitory checkpoint for phagocytosis, playing a crucial role in escaping phagocytic clearance and suppressing innate immune activation across various human malignancies (9, 10). These studies indicate that immune evasion mechanisms may intersect with DNA damage response pathways.

DNA damage responses are orchestrated by two key damage-induced protein kinases: ataxia-telangiectasia mutated (ATM) and ataxia telangiectasia mutated and Rad3-related (ATR) (11). ATR, along with its functional partner ATRIP, is activated in response to various forms of DNA damage and replication stress. This activation is initiated by the exposure of single-stranded DNA (ssDNA), which is coated with Replication Protein A (RPA) (11–13). RPA is a high-affinity ssDNA binding protein complex composed of three subunits: RPA1 (70 kDa), RPA2 (32 kDa), and RPA3 (14 kDa), with RPA1 being as the key component (14–16). Acting as a scaffold to recruit essential DNA repair factors, the RPA complex plays a crucial role in activating DNA damage response signaling and maintaining genome stability (14, 15). ETAA1 (Ewing tumor-associated antigen 1), an RPA -interacting protein, participates in cellular responses to replication stress by activating ATR through a conserved ATR activating domain (AAD). ETAA1 is recruited to sites of DNA damage by interacting with the RPA complex via its RPA -binding domains (11, 17, 18).

In light of this understanding, we hypothesize that the RPA1-ETAA1 axis may play a role in regulating PD-1/PD-L1 signaling in liver cancer, potentially influencing immune surveillance and tumor metastasis. Exploring this axis may uncover an alternative pathway in liver cancer development, providing new insights into potential therapeutic targets.

## Materials and methods

### Tissue array immunohistochemistry

Formalin-fixed paraffin-embedded tissue array sections, including 25 cases of primary hepatocellular carcinomas and adjacent liver tissues (Catalog# HLiv-HCC050PG-01), were obtained from Outdo Biotech (Shanghai, China). These sections were stained with an anti-RPA1 antibody (Biorbyt #orb556563) at a 1:500 dilution. Only cases with technically satisfactory staining were used for further analysis. RPA1 expression levels were evaluated using a combined score of staining intensity and distribution. Staining intensity was classified into four levels: 0 (negative), 1+ (weakly positive), 2+ (moderately positive), and 3+ (strongly positive); The percentage of stained cells was categorized as 0 (0%), 1(< 25%), 2 (25–50%), 3 (50–75%), and 4 (>75%). The total score was calculated by multiplying the intensity score by the corresponding distribution score. A cutoff total score of eight was used to distinguish between cases with high and low RPA1 expression. Images were visualized and exported using Case viewer 2.4 software (3D HISTECH LTD).

### Tumor immune estimation resource analyses

TIMER2.0 is a comprehensive tool that integrates multiple algorithms to investigate various associations between immune cell infiltrates and clinical or genetic features within The Cancer Genome Atlas (TCGA) cohorts (19–21). In this study, we explored correlations between gene markers with a focus on 371 primary Liver hepatocellular carcinoma (LIHC) samples. Spearman’s correlation was visualized using heat maps and scatter plots, and purity-adjusted partial rho coefficients were calculated.

The Gene module was used to examine correlations between RPA1 expression and the abundance of immune infiltrates, such as CD8+ T cells, CD4+ T cells, natural killer T cells, regulatory T cells, neutrophils, cancer -associated fibroblasts (CAFs) and myeloid -derived suppressor cells (MDSCs). Results were presented in scatter plots, with purity-adjusted correlation coefficients and statistical significance.

The Outcome module was utilized to evaluate the impact of immune infiltrating subsets on the clinical outcomes of LIHC patients. Kaplan-Meier curves were generated, and the log-rank test was used to calculate hazard ratios (HR) and p-values for assessing statistical significance.

### Gene correlation analysis using GEPIA

Gene Expression Profiling Interactive Analysis (GEPIA) is a tool that integrates gene expression profiles from both cancer and healthy cohorts, allowing for pairwise gene analysis (22). In this study, we examined correlations between gene markers using the primary LIHC (n=369) and normal liver tissue (n=50) samples from TCGA. Scatter plots were generated to visualize the correlation coefficients and significance.

### Queries of TCGA data via Cbioportal and UALCAN web portal

The cBioPortal for Cancer Genomics provides a web resource for integrative analysis (23–25). Gene expression correlations were explored using the Liver Hepatocellular Carcinoma dataset (TCGA, PanCancer Atlas), with results displayed as scatter plots in Log2 RSEM (normalized from RNASeqV2) (n=366). Both Spearman’s and Pearson’s coefficients, along with their significance levels, were also indicated.

UALCAN integrates data from TCGA and CPTAC to compare primary tumors with normal tissue samples and further analyze different tumor subgroups based on clinical parameters, such as cancer stages and tumor grades (26–30). In this study, we examined 165 primary LIHC and 165 normal liver tissue samples from CPTAC to assess RPA1 protein expression. The protein levels were log2 normalized and presented as Z-values. To analyze the clinicopathologic characteristics of RPA1 and ETAA1 in LIHC, we compared the primary LIHC samples with normal liver tissues from the TCGA cohorts. Cancer stages were categorized as Stage I, II, III, and IV, while tumor grades were classified as well differentiated (Grade 1), moderately differentiated (Grade 2), poorly differentiated (Grade 3), and undifferentiated (Grade 4).

### STRING database

The STRING database provides access to predicted functional protein-protein interactions (PPI) (31). The PPI network for Homo sapiens was constructed using STRING v12.0, with all active interaction parameters selected, including text-mining, experiments, databases, co-expression, neighborhood, gene fusion, and co-occurrence. The STRING interactants was summarized, yielding a PPI enrichment p-value of 1.32 × 10^−8^.

### Estimation of infiltrating immune cells by ESTIMATE algorithm

ESTIMATE (Estimation of STromal and Immune cells in MAlignant Tumor tissues using Expression data) is used to calculate immune enrichment scores to infer the levels of infiltrating immune cells (32). ETAA1 RNASeqV2 data were collected from the TCGA PanCancer Atlas cohort via cBioPortal. The corresponding immune scores were obtained from the RNASeq V2 platform using ESTIMATE, and were compared based on the differential ETAA1 expression levels.

### Statistical analyses

**Figure 1:**
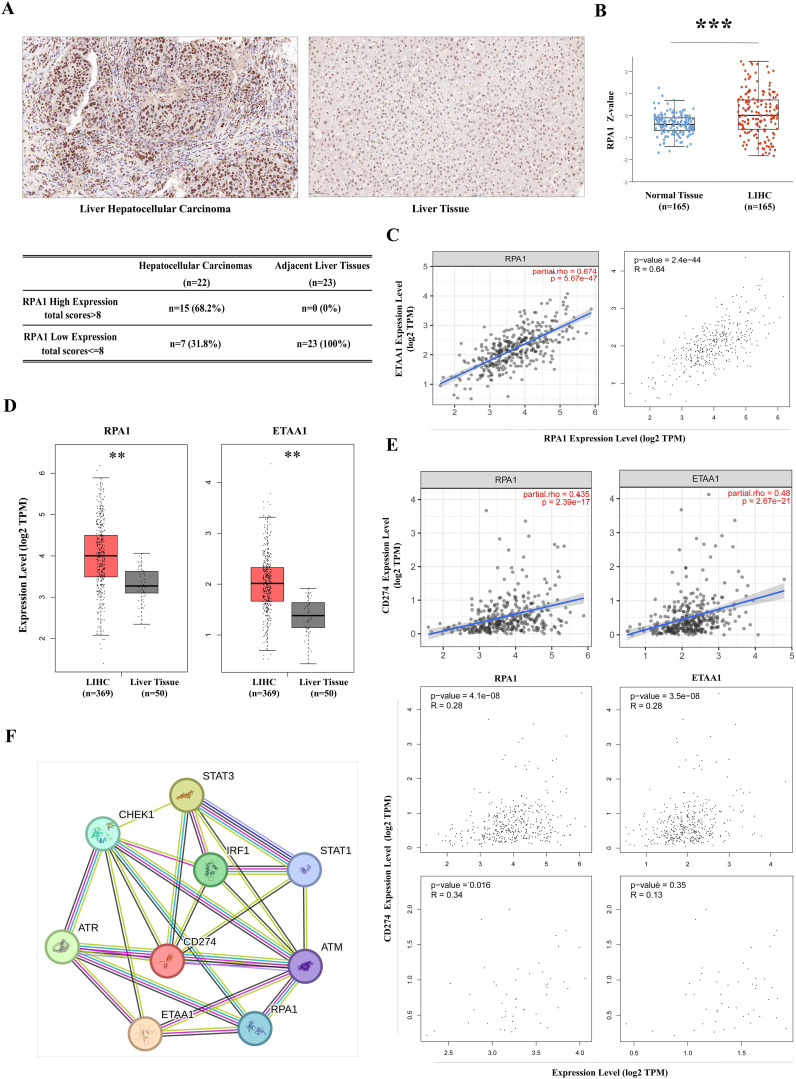
RPA1-ETAA1 Association Linked to PD-L1 in Hepatocellular Carcinoma **(A)** Immunohistochemistry for RPA1 was performed on primary hepatocellular carcinoma cases and adjacent liver tissues. Representative images of RPA1 expression are shown for hepatocellular carcinoma (*Left*) and adjacent tissues (*Right*) at a scale of 50 μm. Expression levels were evaluated using a combined score based on staining intensity and distribution, Each sample was assigned a total score. A cutoff total score of eight was used to distinguish between cases with high and low RPA1 expression (*Lower*) **(B)** RPA1 protein levels in primary LIHC and normal liver tissue samples were analyzed using CPTAC cohorts. Expression levels are log2 normalized and presented as Z-values *** p<0.001. **(C)** The correlation between RPA1 and ETAA1 in LIHC samples was analyzed using TIMER2 (*Left*) and GEPIA (*Right*). **(D)** RPA1 and ETAA1 expression levels were analyzed using UALCAN, with box plots showing expression levels in primary LIHC (n = 369) and normal liver tissue samples (n = 50). ** p<0.01. **(E)** Correlations of RPA1 and ETAA1 with PD-L1 in LIHC samples were analyzed using TIMER2 (*Upper*) and GEPIA (*Middle*). Correlations in normal liver tissues were also examined using GEPIA (*Lower*) **(F)** The PPI network involving RPA1, ETAA1, and PD-L1 was constructed using STRING v12.0, and all potential interacting partners were summarized.

**Figure 2:**
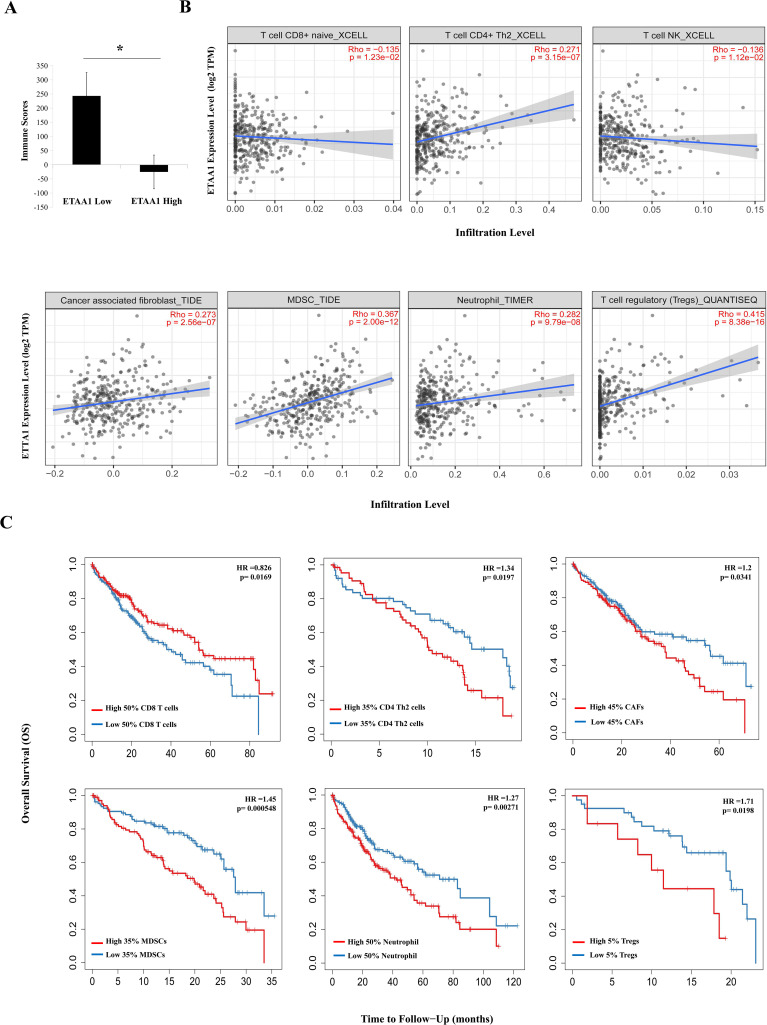
ETAA1 Shapes Immunophenotypes in Liver Hepatocellular Carcinoma. **(A)** Immune scores from the ESTIMATE algorithm were compared between high and low ETAA1 expression levels.**(B)** In LIHC (n = 371), the correlations between ETAA1 and the infiltration levels of CD8+ T cells, CD4+ Th2 cells, NK T cells, Tregs, neutrophils, CAFs and MDSCs were analyzed using the *Gene* module of TIMER2.0, with Spearman’s rho and statistical significance indicated. **(C)** Kaplan-Meier curves were generated to visualize the differences in survival rates between patients with high versus low infiltration levels of these immune cells. The hazard ratio (HR) and statistical significance were calculated using the log-rank test.

**Figure 3:**
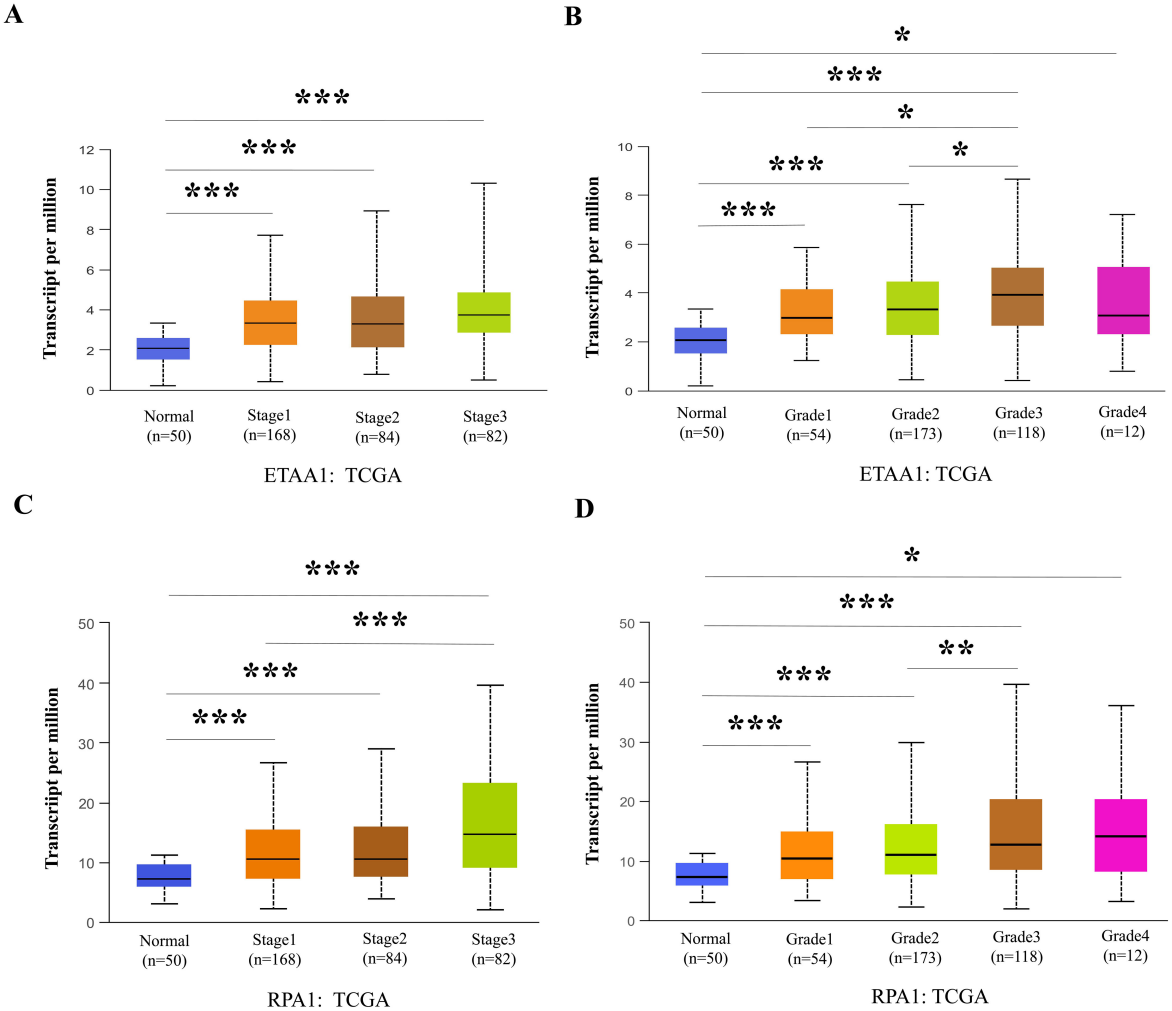
RPA1-ETAA1 Axis Links to Unfavorable Progression in Liver Cancer. UALCAN analysis revealed ETAA1 levels across liver cancer stages **(A)** and tumor grades **(B)**, as well as RPA1 levels across liver cancer stages **(C)** and tumor grades **(D)**. Expression levels were presented as transcripts per million (TPM). *P < 0.05, **p<0.01, ***P < 0.001.

**Figure 4:**
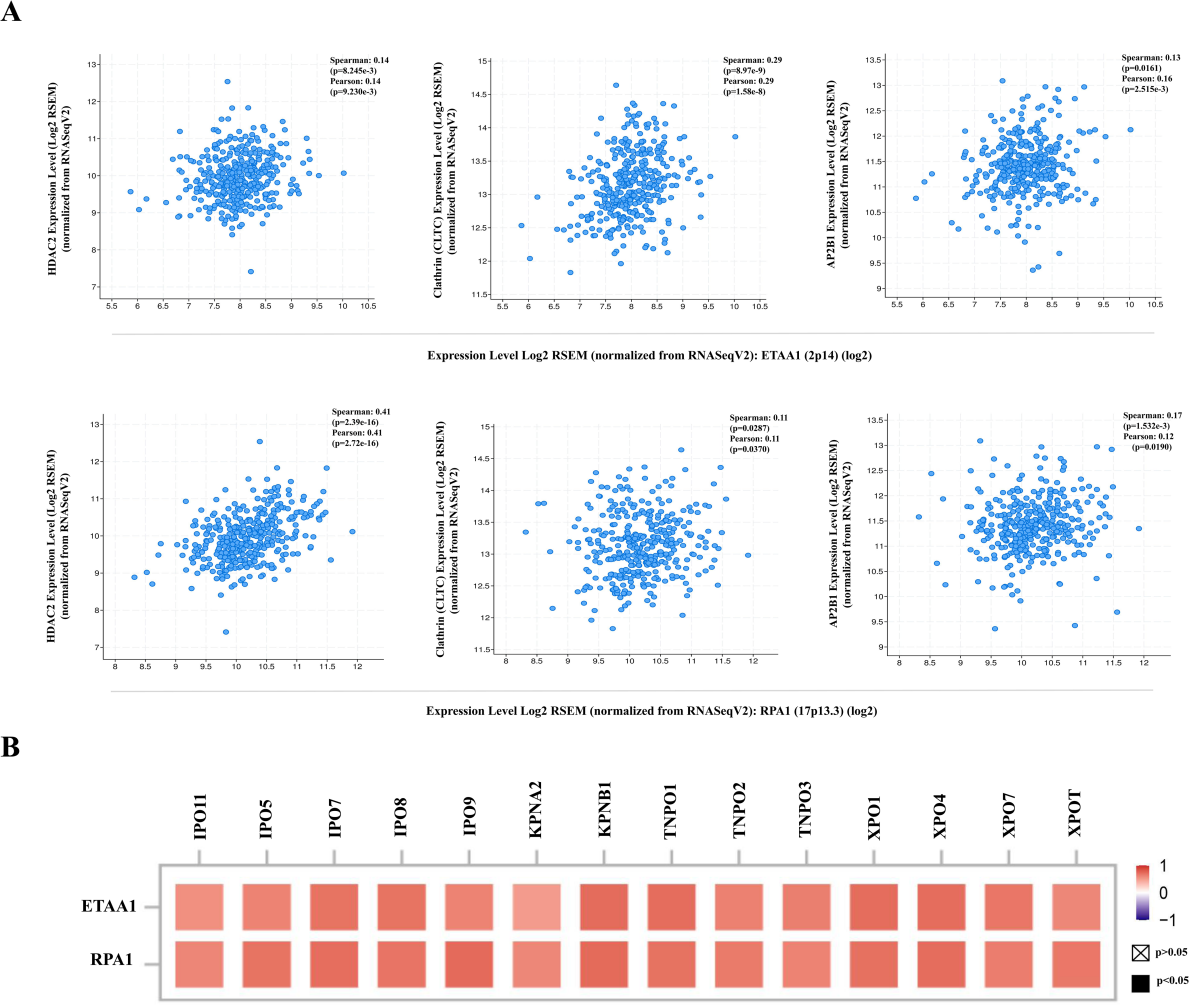
RPA1-ETAA1 Axis Correlates with PD-L1 Nuclear Translocation. **(A)** The correlations of ETAA1 and RPA1 with key regulators of PD-L1 nuclear translocation, including HDAC2 (*Left*), clathrin (CLTC) (*Middle*), and AP2B1 (*Right*) were analyzed in LIHC samples using cBioPortal. **(B)** The correlations of ETAA1 and RPA1 with key importins, transportins, and exportins involved in PD-L1 nuclear translocation were analyzed using TIMER2 and presented in a heatmap.

Chi-square and Fisher’s exact tests were used to analyze the differential expression of RPA1 in the tissue array samples shown in **Figure 1A**; Expression levels were compared using an unpaired t-test in **Figures 1B, D**, **2A**, **3**. Spearman’s or Pearson’s correlation analyses were conducted in **Figures 1C, E**, **4**, **2B**, as well as in **Tables 1**–**4**. Survival analysis in **Figure 2C** was performed using log-rank tests. Statistical significance is indicated as follows: * p<0.05, ** p<0.01, *** p<0.001.

**Table 1:** Correlation Analysis of ETAA1-RPA1 with Regulators of PD-L1 Nuclear Translocation in LIHC via TIMER2.0.

Gene Markers			ETAA1		RPA1	
			Purity-adjusted partial spearman's rho	p Value	Purity-adjusted partial spearman's rho	p Value
**PD-L1 Nuclear Translocation**	**Importins**	Importin 11 (IPO 11)	0.554	***	0.605	***
		Importin 5 (IPO 5)	0.62	***	0.694	***
		Importin 7 (IPO 7)	0.693	***	0.717	***
		Importin 8 (IPO 8)	0.697	***	0.688	***
		Importin 9 (IPO 9)	0.616	***	0.735	***
		Importin α1 (KPNA2)	0.493	***	0.597	***
		Importin β1 (KPNB 1)	0.728	***	0.737	***
	**Transportins**	Transportin 1 (TNPO1)	0.722	***	0.701	***
		Transportin 2 (TNPO2)	0.627	***	0.657	***
		Transportin 3 (TNPO3)	0.636	***	0.619	***
	**Exportins**	Exportin 1 (XPO1)	0.722	***	0.703	***
		Exportin 4 (XPO4)	0.722	***	0.722	***
		Exportin 7 (XPO7)	0.676	***	0.646	***
		Exportin T (XPOT)	0.597	***	0.672	***

*** p<0.001.

## Results

### RPA1-ETAA1 association linked to PD-L1 in hepatocellular carcinoma

High tumor mutational burden (TMB) in malignancies reflects an accumulation of DNA replication errors and mutations that evade immune surveillance and elimination (33). This genomic instability may be driven by excessive production of peptide neoantigens, contributing to the upregulation of the PD-1/PD-L1 axis (33). ATR is activated in response to various forms of DNA damage and replication stress, initiated by the exposure of ssDNA coated with the RPA complex. As a crucial member of this complex, RPA1, when dysfunctional, can lead to genomic instability and the development of lymphoid tumor (34). Therefore, we began our investigation with a pan-cancer screening of RPA1 using TCGA data, which revealed a significant up-regulation of RPA1 in liver hepatocellular carcinoma (LIHC) compared to adjacent normal tissue controls (p<0.001) (**Supplementary Figure 1**). DNA damage and repair are particularly critical in the liver primarily due to its extensive involvement in metabolism, making it susceptible to oxidative damages (35). A tissue array confirmed elevated RPA1 expression and enhanced nuclear localization in primary hepatocellular carcinoma samples (**Figure 1A**). By taking into consideration of staining intensity and distribution, a total score was assigned to each sample. With a cutoff score of eight, 68.2% of the hepatocellular carcinoma samples exhibited high RPA1 expression, while none of the adjacent liver tissues showed high expression (p<0.001). Further validation using CPTAC cohorts demonstrated a substantial upregulation of RPA1 protein levels in LIHC (p<0.001) (**Figure 1B**). As expected, a strong positive correlation between RPA1 and ETAA1 was observed in TIMER2 and GEPIA analyses (p<0.001) (**Figure 1C**). Both RPA1 and ETAA1 were coordinately upregulated in LIHC (p<0.01) (**Figure 1D**). Importantly, significant associations between RPA1, ETAA1 and PD-L1 were identified (p<0.001), with these connections being specific to the tumor context (**Figure 1E**). While the relationships among these three molecules are emerging, expanding this network is necessary to find additional valuable interactive partners for our exploration. To this end, we used a predicted functional PPI network, which preliminarily indicated potential interactors involved in DNA damage responses and ATR pathway activities (**Figure 1F**). This underscores the need for an in-depth investigation into how these interactions may influence PD-L1 actions.

### RPA1-ETAA1 axis correlates with PD-L1 nuclear translocation

The deacetylation of PD-L1 by histone deacetylase 2 (HDAC2) has been reported to promote its nuclear translocation through interactions with various proteins involved in endocytosis and nucleocytoplasmic transport (6). Once within the nucleus, PD-L1 regulates the expression of multiple immune response-related genes, thereby shaping tumor-immune dynamics (6). To further elucidate how ETAA1 and RPA1 may impact PD-L1’s nuclear functions, we examined their relationship with key components of these pathways. Our analysis revealed a positive correlation between the expression levels of ETAA1 and its binding partner RPA1 with HDAC2 in LIHC (**Figure 4A** *left* and **Supplementary Figure 2A** *left*). Coordinated up-regulation of HDAC2 protein was also observed in the LIHC samples (**Supplementary Figure 2B**).

PD-L1 intracellular trafficking is initiated through Clathrin -dependent endocytosis, wherein Clathrin (CLTC) selectively binds to cargo adaptors, particularly via Adaptin β2 (AP2B1), to tether unacetylated PD-L1 to Clathrin (6, 36). Our analysis revealed strong correlations between ETAA1 and RPA1 with both Clathrin and AP2B1, indicating their potential involvement in PD-L1 transport (**Figure 4A**; **Supplementary Figure 2A**). Following this, PD-L1’s nuclear import hinges on interactions with importin α family members, which have been recognized as PD-L1-interacting proteins (6, 37). We further examined the relationships between ETAA1, RPA1, and these nuclear transport receptors, discovering significant associations linked to PD-L1 nucleocytoplasmic shuttling (**Figure 4B**; **Table 1**). Within the nucleus, PD-L1 transactivates a panel of pro-inflammatory and immune response transcription factors, including those involved in the IRF, JAK - STATs and NF-κB signaling pathways (6, 8). To explore these potential interactions, we screened representative genes from these pathways. Beyond verifying their known associations with PD-L1, our analyses further revealed connections between these genes and both ETAA1 and RPA1, as detailed in **Table 2**. These suggest that the activation of these pathways may be attributed to RPA1-dependent, ETAA1-mediated ATR activity, which, in turn, drives PD-L1 upregulation.

**Table 2:** Correlation Analysis of PD-L1, ETAA1, and RPA1 with Nuclear Signaling Regulators using TIMER2.0

Gene Markers	PD-L1 (CD274)		ETAA1		RPA1	
	Purity-adjusted partial spearman's rho	*P* Value	Purity-adjusted partial spearman's rho	*P* Value	Purity-adjusted partial spearman's rho	*P* Value
IRF1	0.607	***	0.49	***	0.431	***
STAT1	0.444	***	0.512	***	0.517	***
STAT3	0.452	***	0.448	***	0.411	***
JAK1	0.552	***	0.635	***	0.559	***
JAK2	0.731	***	0.692	***	0.641	***
TYK2	0.449	***	0.566	***	0.572	***
NF-κB p50	0.545	***	0.642	***	0.669	***
NF-κB p52	0.164	**	0.264	***	0.298	***
RELA p65	0.422	***	0.532	***	0.597	***

** p<0.01, *** p<0.001.

**Figure 5:**
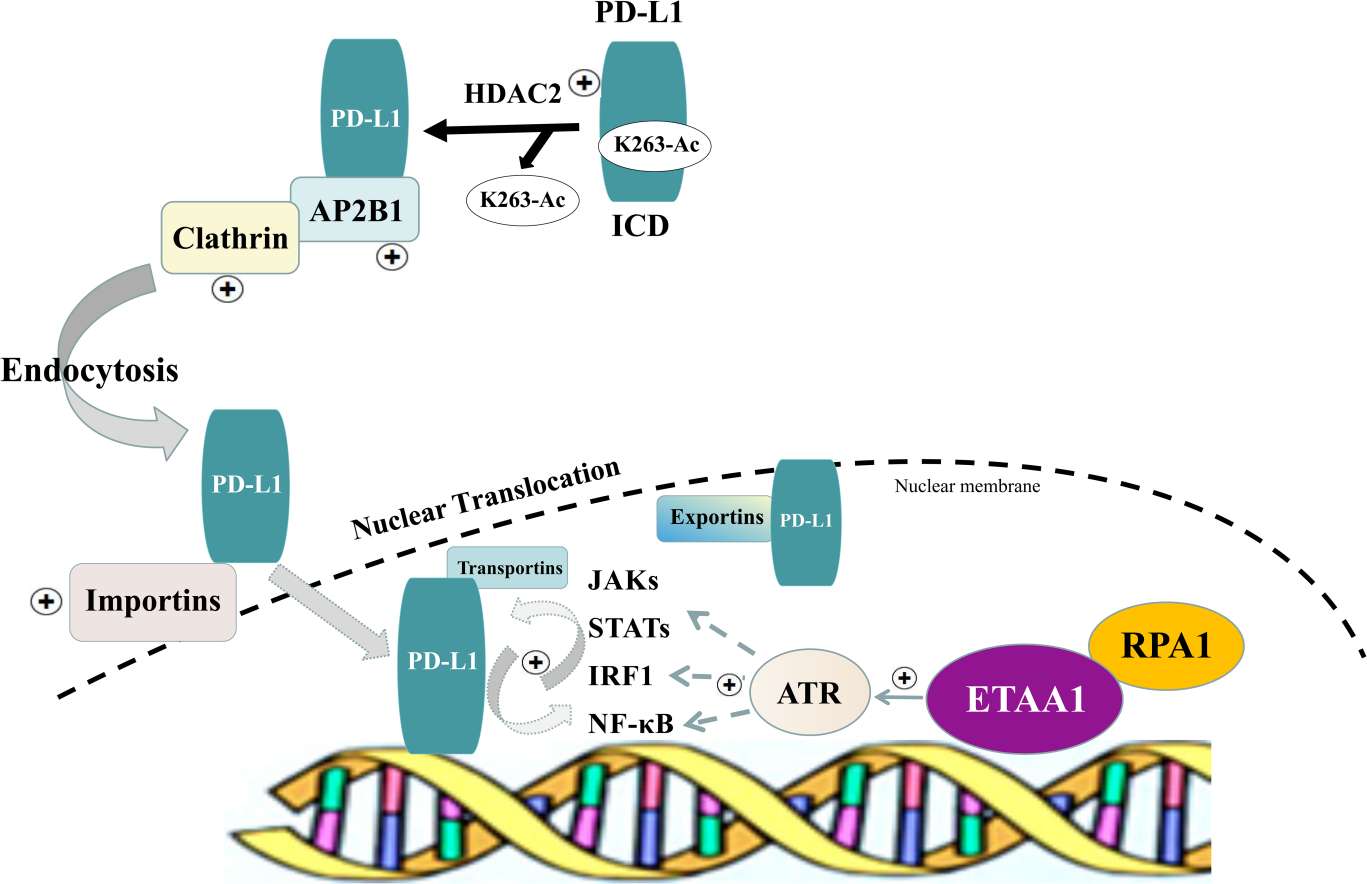
Schematic of RPA1-ETAA1 Axis Mediated PD-L1 Nuclear Translocation. A model illustrating RPA1-dependent, ETAA1-mediated PD-L1 nuclear translocation. Upregulation of RPA1 enhances ETAA1 recruitment, subsequently activating ATR signaling pathways. This facilitates PD-L1 nuclear translocation through modulating several processes, including HDAC2-mediated deacetylation, clathrin-dependent endocytosis, nucleocytoplasmic shuttling and nuclear import of PD-L1. Within the nucleus, PD-L1 transactivates the IRF, JAK - STATs and NF-κB signaling pathways, which are also influenced by ATR activation, creating a positive feedback loop that further increases nuclear PD-L1 levels.

In light of these findings, we propose a model in which the RPA1-ETAA1 axis mediates tumor-immune interactions by influencing PD-L1 nuclear translocation (**Figure 5**). In this model, upregulated RPA1 enhances the recruitment of ETAA1, leading to the activation of ATR signaling pathways. This facilitates PD-L1 nuclear translocation by modulating several processes, including HDAC2-mediated deacetylation, clathrin-dependent endocytosis, PD-L1 nucleocytoplasmic shuttling and nuclear import. Within the nucleus, PD-L1 transactivates multiple pro-inflammatory and immune response transcription factors that are also regulated by ATR activation, building up a positive feedback loop that further increases nuclear PD-L1 expression and foster tumor immune evasion.

### ETAA1 shapes immunophenotypes in liver hepatocellular carcinoma

Considering the potential interplay between PD-L1 translocation and tumor invasiveness, we next investigated the role of ETAA1 in this context, revealing a diverse landscape of tumor infiltrating lymphocytes (TILs) within the tumor microenvironment (TME) engaged by ETAA1. As immune signatures within the TME are predictive of tumor invasiveness and metastasis, we evaluated the impact of ETAA1 on the enrichment of various immune cell populations. Our results demonstrated a negative immune score that inversely correlated with ETAA1 expression (**Figure 2A**). To further delineate the cellular composition of the infiltrates influenced by ETAA1 in LIHC, we performed correlation analyses between ETAA1 and individual cell subsets. We found that levels of ETAA1 were negatively correlated with naive CD8+ T (Spearman’s rho = −0.135, p < 0.05) and NKT cells (Spearman’s rho = −0.136, p < 0.05), while showing positive correlations with CD4 + Th2 cells (Spearman’s rho =0.271, p < 0.001), CAFs (Spearman’s rho =0.273, p < 0.001), MDSCs (Spearman’s rho = 0.367, p < 0.001), neutrophils (Spearman’s rho =0.282, p < 0.001) and Tregs (Spearman’s rho = 0.415, p < 0.001) (**Figure 2B**). Although the roles of some of these cell types are established, we also evaluated their associations with clinical outcomes in LIHC. Our analysis revealed that elevated levels of CD4 + Th2 cells (p < 0.05), CAFs (p<0.05), MDSCs (p < 0.001), Neutrophils (p < 0.01) and Tregs (p<0.05) were significantly associated with poorer overall survival (OS) rates. In contrast, higher levels of CD8+ T cells were associated with improved OS in LIHC (**Figure 2C**). In addition to analyzing the major cellular components of the infiltrates, we also examined the relationship between ETAA1 expression and specific gene representatives characteristic of these infiltrating cells. Our results, summarized in **Table 3**, indicated significant associations between ETAA1 and these metagenes in LIHC, which were further validated using GEPIA (**Table 3**). These findings support potential actions of ETAA1 in tumor- immune interactions, which may contribute to an alternate escaping mode of PD-L1 against host immunity.

**Table 3:** Correlation Analysis of ETAA1 with Gene Markers of Immune Cell Subsets using TIMER2.0 and GEPIA.

Liver Hepatocellular Carcinoma (LIHC)					
Cell Types	Gene Markers	ETAA1 Correlation (TIMER 2.0)		ETAA1 Correlation (GEPIA)	
		Purity-adjusted partial spearman's rho	*P* Value	Spearman's R	*P* Value
**CD8 T cells**	CD8A	0.274	***	0.15	**
**CD4+ Th2 cells**	GATA3	0.381	***	0.24	***
	STAT6	0.415	***	0.43	***
**CAFs**	CD44	0.275	***	0.12	*
	FAP	0.346	***	0.16	**
	PDGFRα	0.317	***	0.16	**
	PDGFRβ	0.361	***	0.24	***
**MDSCs**	CD11b (ITGAM)	0.405	***	0.36	***
	IL-4R	0.439	***	0.43	***
	CD33	0.283	***	0.12	*
**Regulatory T cells (Tregs) & T cell Exhaustion**	FOXP3	0.363	***	0.16	**
	PD-1 (PDCD1)	0.211	***	0.14	**
	CTLA4	0.185	***	0.12	*
	TGFβ (TGFB1)	0.359	***	0.17	**
	IL-10	0.294	***	0.17	**
	TIM-3 (HAVCR2)	0.391	***	0.23	***
	ITGA4	0.643	***	0.45	***
	PTGIR	0.235	***	0.11	*
	STAT5B	0.587	***	0.57	***
	L1CAM	0.302	***	0.26	***
**Neutrophils**	CCL2	0.301	***	0.15	**
	CXCL1	0.233	***	0.19	***
	IL-8 (CXCL8)	0.321	***	0.22	***
	CXCL16	0.149	**	0.1	*

* p<0.05, ** p<0.01, *** p<0.001.

### RPA1 - ETAA1 axis links to metastasis mediators and unfavorable progression in liver cancer

Nuclear PD-L1 transactivates multiple genes involved in regulating cancer invasiveness and metastasis through various pathways, including epithelial- mesenchymal transition (EMT) (38–40). To further investigate this relationship, we assessed the association of ETAA1 and RPA1 with these markers, revealing significant correlations as summarized in **Table 4**. Furthermore, we explored the clinicopathologic characteristics of RPA1-dependent, ETAA1-mediated events in liver cancer patients by analyzing the correlations between ETAA1 and RPA1 expression levels and liver cancer stages and tumor grades. UALCAN analysis indicated a preferential up-regulation of ETAA1 and RPA1 in advanced liver cancer stages (**Figures 3A, C**) and in poorly differentiated tumor grades (**Figures 3B, D**), when compared to normal tissue controls. These findings underscore the potential of ETAA1 and RPA1 as indicators of liver cancer progression, highlighting their clinical relevance as targets for therapeutic intervention.

**Table 4:** Correlation Analysis of RPA1-ETAA1 with Nuclear Factors Linked to Tumor Metastasis using TIMER2 and GEPIA.

Gene Markers	ETAA1 Correlation (TIMER 2.0)		ETAA1 Correlation (GEPIA)		RPA1 Correlation (TIMER 2.0)		RPA1 Correlation (GEPIA)	
	Purity-adjusted partial spearman's rho	*P* Value	Spearman's R	*P* Value	Purity-adjusted partial spearman's rho	*P* Value	Spearman's R	*P* Value
EGFR	0.504	***	0.46	***	0.381	***	0.36	***
HIF1α	0.677	***	0.57	***	0.629	***	0.6	***
Snail (SNAI1)	0.258	***	0.11	*	0.207	***	0.15	**
Slug (SNAI2)	0.226	***	0.19	***	0.16	**	0.19	***
Twist (TWIST1)	0.317	***	0.25	***	0.339	***	0.34	***
Jun	0.31	***	0.28	***	0.283	***	0.3	***
Fos	0.238	***	0.15	**	0.189	***	0.18	***
IFNγ	0.179	***	0.12	*	0.233	***	0.18	***
IL-6	0.234	***	0.13	*	0.187	***	0.14	**
TNFα	0.573	***	0.21	***	0.445	***	0.18	***

* p<0.05, ** p<0.01, *** p<0.001.

## Discussion

In response to replication stress and DNA double-strand breaks (DSBs), ETAA1 is recruited to DNA damage sites through its dual RPA -binding and ATR activating domains (AAD) motifs, functioning as an ATR activator within ATR -ATRIP complexes. This facilitates the phosphorylation and activation of downstream effectors, such as Chk1, halting the cell cycle to allow DNA repair (17). RPA binding is crucial for ETAA1’s regulation of ATR activity. RPA1 is key to maintaining genomic stability and regulating tumor cell responses to genotoxic stress from treatments like chemotherapy or radiation (8, 17). The RPA1-ETAA1 axis supports cellular survival in response to DNA damage and may also contribute to immune evasion through PD-L1 upregulation, enabling tumor cells to evade immune surveillance in stressed environments. Our findings associate PD-L1 with ETAA1 and RPA1, suggesting that the upregulated RPA1-ETAA1 axis may potentially support PD-L1 nuclear accumulation through HDAC2-mediated deacetylation, clathrin-dependent endocytosis, and PD-L1 nucleocytoplasmic shuttling. This nuclear accumulation of PD-L1 may subsequently activate a panel of pro-inflammatory and immune response transcription factors. These signal cascades may reshape tumor immune microenvironment and facilitate the infiltration of various lymphocytes associated with clinical outcomes in LIHC, thereby contributing to the immune suppressive microenvironment and immune evasion observed in this setting. To ensure a comprehensive collection of patient cases, we leveraged open-source data from major cancer projects like TCGA and CPTAC to explore the interactive expression patterns of ETAA1 and RPA1 with PD-L1, as well as multiple correlations with the mediators of PD-L1 nuclear translocation and PD-L1 associated nuclear transcriptional factors. This approach allowed us to overcome limitations in our own sample collection.

Our hypothesis is supported by prior evidence that RPA1-dependent, ETAA1-mediated ATR signaling activates transcription factors or signaling molecules such as NF-κB or JAK - STATs, enhancing PD-L1 activity (8) and linking DNA repair mechanisms to immune checkpoint regulation. Additionally, the nuclear accumulation of PD-L1 can transactivate a range of pro-inflammatory and immune response transcription factors involved in the IRF, JAK - STATs and NF-κB signaling pathways (6), creating a positive feedback loop that further enhances nuclear PD-L1 expression and fosters tumor immune evasion. The activation of these signaling pathways in response to chemotherapy and radiation also results in PD-L1 upregulation, contributing to treatment resistance. Therefore, targeting the RPA1-ETAA1 axis alongside immune checkpoint inhibitors may offer promising therapeutic strategies for improving clinical outcomes. We remain committed to elucidating the specific factors involved in this regulatory network and their implications for therapeutic resistance and immune evasion.

Liver cancer, recognized as an immunogenic malignancy, is characterized by an intrinsically immune-suppressive microenvironment and high levels of immune evasion (41). Our findings indicate that elevated levels of Tregs, MDSCs and CAFs, which secrete inhibitory cytokines, correlate with poor prognosis. Moreover, the infiltration of CD4^+^ Th2 cells and Neutrophils is linked to adverse outcomes, as demonstrated by survival analysis. Although CD8^+^ T cells and NK T cells exhibit significant anti-tumor potential through secreting pro-inflammatory cytokines that enhance anti-tumor responses, their functions are frequently suppressed by various immunosuppressive mechanisms (42, 43). In this context, we identified a landscape of infiltrating lymphocytes influenced by ETAA1. Our analysis revealed a negative correlation between ETAA1 levels and CD8+ T and NKT cells, while showing a positive correlation with CD4+ Th2 cells, CAFs, MDSCs, neutrophils, and Tregs. These significant correlations suggest that ETAA1 may serve as a potential indicator of immune evasion. Patients with high ETAA1 expression may benefit less from immunotherapies that rely on functional CD8+ T and NK T cells, such as immune checkpoint inhibitors. Thus, evaluating ETAA1 expression could aid in patient stratification and contribute to personalized treatment strategies.

Our investigation into the RPA1-ETAA1 axis aims to assess the clinicopathological characteristics of liver cancer patients to customize treatment protocols and provide precise prognostic insights. Patients with elevated expression of RPA1 and ETAA1 may be considered as high-risk, necessitating more aggressive treatment or intensified monitoring protocols. Our findings highlight a novel role for the RPA1-ETAA1 association that extends beyond its established function in DNA repair regulation. Although still in the preliminary stages, these insights offer a promising framework for future studies exploring the molecular mechanisms of RPA1-dependent ETAA1 in cancer interventions. Ultimately, this research could pave the way for more efficacious targeting strategies in liver cancer therapy.

## Data Availability

The original contributions presented in the study are included in the article/**Supplementary Material**. Further inquiries can be directed to the corresponding author.

## Supplementary material

Supplementary Figure 1 Human RPA1 expression levels were analyzed across various tumor types in the TCGA dataset using the *Gene_DE* module of TIMER2. Red bars represent the expression levels in tumors, while blue bars indicate the expression levels in adjacent normal tissues. Up- or down- regulation of RPA1 is denoted by the symbols (*P < 0.05, **P < 0.01, and ***P < 0.001).

Supplementary Figure 2
**(A)** The correlations of ETAA1 and RPA1 with key regulators of PD-L1 nuclear translocation, including HDAC2, clathrin (CLTC), and AP2B1 in LIHC samples were analyzed using TIMER2. **(B)** Protein levels of HDAC2 in primary LIHC and normal liver tissue samples were analyzed using the CPTAC cohorts. Expression levels are log2 normalized and presented as Z-values.
