# Supplementary figures and images for: Exploring RPA1-ETAA1 axis via high-throughput data analysis: implications for PD-L1 nuclear translocation and tumor-immune dynamics in liver cancer
Source: Front Immunol. 2024 Nov 26;15:1492531. doi: 10.3389/fimmu.2024.1492531 (PMC11628550; doi:10.3389/fimmu.2024.1492531)

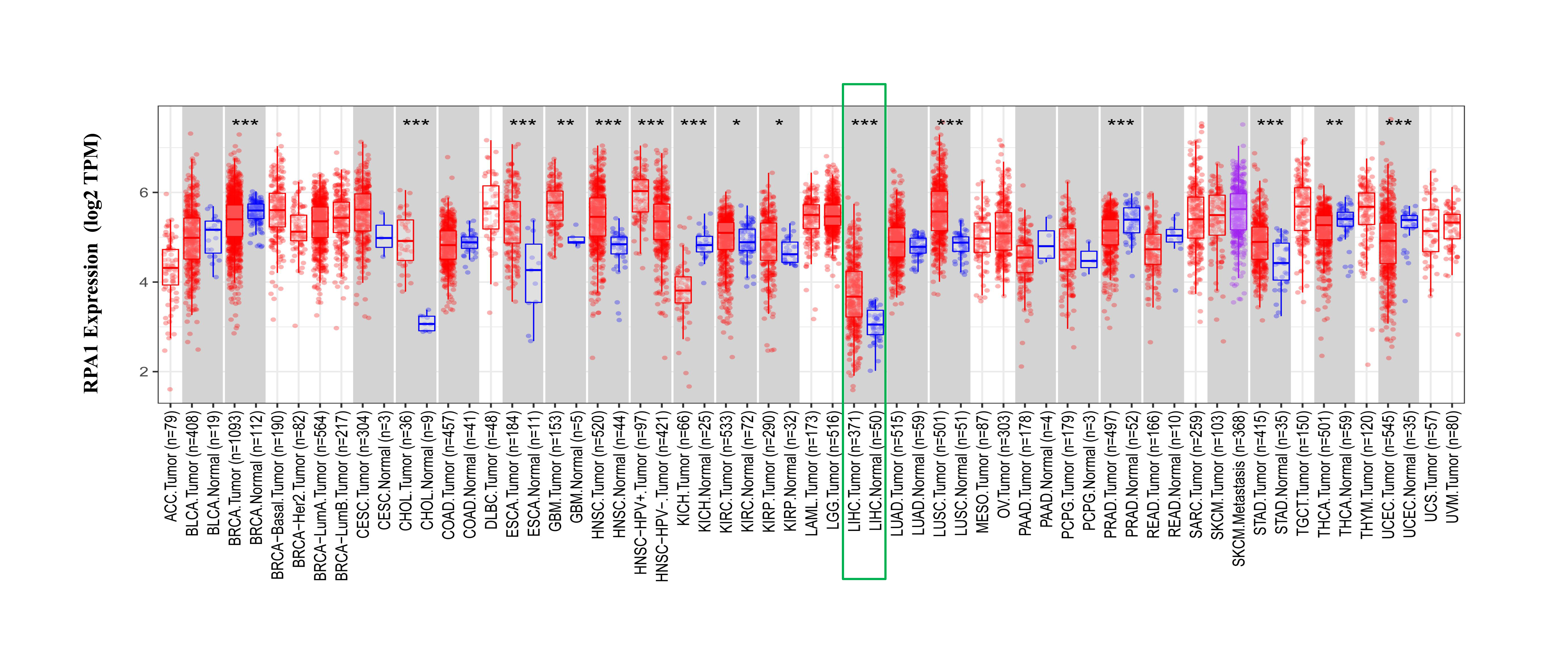

Supplement: Supplementary Figure 1 — Human RPA1 expression levels were analyzed across various tumor types in the TCGA dataset using the Gene_DE module of TIMER2. Red bars represent the expression levels in tumors, while blue bars indicate the expression levels in adjacent normal tissues. Up- or down- regulation of RPA1 is denoted by the symbols (*P < 0.05, **P < 0.01, and ***P < 0.001). [file Image1.tif]

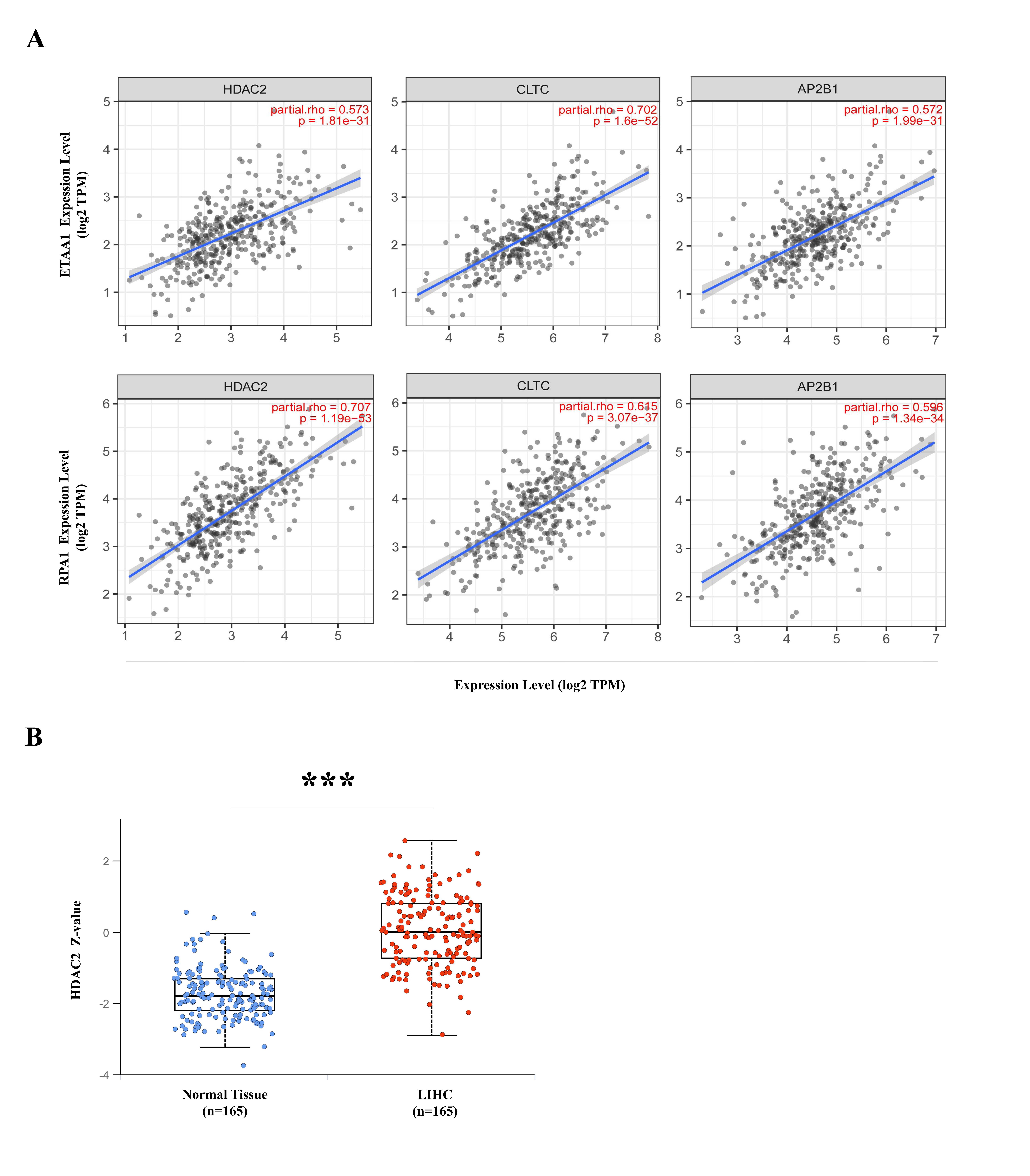

Supplement: Supplementary Figure 2 — (A) The correlations of ETAA1 and RPA1 with key regulators of PD-L1 nuclear translocation, including HDAC2, clathrin (CLTC), and AP2B1 in LIHC samples were analyzed using TIMER2. (B) Protein levels of HDAC2 in primary LIHC and normal liver tissue samples were analyzed using the CPTAC cohorts. Expression levels are log2 normalized and presented as Z-values. [file Image2.tif]
